# Teacher Engagement in Language Teaching: Investigating Self-Efficacy for Teaching Based on the Project “*Sino-Greece Online Chinese Language Classrooms”*

**DOI:** 10.3389/fpsyg.2021.710736

**Published:** 2021-08-04

**Authors:** Chunrong Bao, Lawrence Jun Zhang, Helen R. Dixon

**Affiliations:** ^1^School of Foreign Language Education, Jilin University, Changchun, China; ^2^Faculty of Education and Social Work, The University of Auckland, Auckland, New Zealand

**Keywords:** self-efficacy, CFL teacher, teacher engagement, online teaching, narrative inquiry, case study

## Abstract

The support of sustainable learning of foreign languages requires teacher engagement and a high level of self-efficacy, both of which are cornerstones for the persistence of teachers in carrying out teaching activities to help learning. The need for such attributes is even more crucial when online learning platforms as a mode of delivery are becoming increasingly popular. We would argue that keeping students engaged and motivated to attain their academic success online calls for the increased levels of resilience and efforts of teachers. Although self-efficacy of teachers has been widely considered crucial in the professional practices of teachers, there is a paucity of research studies on the self-efficacy of teachers who teach Chinese as a foreign language (CFL) using online platforms. Such a gap becomes prominent after the sudden outbreak of the COVID-19 pandemic, especially in places where there are now numerous calls for online CFL classes. In order to fill in this gap, this study was conducted with a frontline CFL teacher as the participant and aimed to detect thoroughly the trajectories of self-efficacy of a CFL teacher in a completely new teaching context. Embedded in the Project of Sino-Greece Online Chinese Language Classrooms, this study employed narrative inquiry and case study as methodological approaches. Thematic analysis was used to analyze the data that consisted of written narratives (the teacher's teaching journals and reflections, field notes of teaching assistant, and emails of students) and spoken narratives. Three research questions guided this study: *What are the teacher's beliefs about (1) the opportunity of teaching CFL online? (2) the management of this online project? (3) her personal capability to foster students' engagement in this project?* These three questions focused, respectively, on the three components of the self-efficacy system of a teacher (personal efficacy, efficacy within the organization, and professional efficacy). Findings illustrated that the efficacy beliefs of the teacher in these three aspects were at different levels, which resulted from the interplay of external and internal factors; when external factors appeared to be negative, internal factors seemed to play an essential role.

## Introduction

While the development of the internet has fostered online teaching in the past two decades (Feghali et al., 2021), it is since the sudden outbreak of COVID-19 that online teaching and learning have become a central topic for investigation. As a result, research studies on teaching approaches (Banack et al., 2020; González-Lloret, 2020), supportive technology and resources (Adam, 2020), teacher-student collaboration (Feghali et al., 2021; Li and Zhang, 2021), and cognitions of teachers (Gao and Zhang, 2020; Sun and Zhang, 2021; Wang and Zhang, 2021) have emerged.

However, recent studies have put emphasis on the formidable challenges occurring in the age of COVID-19. However, it would seem that online teaching may occur in the longer term as it becomes a long-lasting, preferred delivery mode (González-Lloret, 2020). In its favor, online delivery provides students with learning resources directly; therefore, students have more choices of learning outside their schools or universities. It can also create a virtual community that bridges the teacher-student geographical distance and embraces various cultures so that both teachers and students can communicate conveniently and have a sense of belonging (Du et al., 2010; González-Lloret, 2020). In addition, online teaching provides learning opportunities for a more diverse range of students, from young students to those who are employed full time, married, or parents (Feghali et al., 2021). As long as highly motivated, any person can be enrolled in online courses to pursue specific learning goals (Feghali et al., 2021).

All of the aforementioned advantages of online teaching have emerged in the field of teaching Chinese as a foreign language (CFL), especially during the COVID-19 pandemic. The pandemic has not drowned the enthusiasm of CFL learners around the world; instead in 2021, more than 180 countries and regions all over the world carried out Chinese language instruction, with over 70 countries having incorporated Chinese into their national education systems, and the number of CFL learners has exceeded 20 million^1^ To satisfy the increasing needs of CFL learners, online CFL courses (e.g., the Sino-Greece Project in this study) have emerged and gained increasing popularity. Yet there is still a paucity of research studies on the potential challenges in online CFL teaching and the emotional states of CFL teachers in having to deal with these challenges. In addition, the available research studies on student engagement were often conducted against the backdrop of offline classrooms. The existing knowledge of behaviors of teachers to foster student engagement cannot solve the problems arising from online teaching. This study, therefore, aimed to address these gaps with a case study of a CFL teacher through using the theoretical lens of self-efficacy.

## Literature Review

### Student Engagement

“Student engagement” is a multifaceted and complex construct (Ashwin and McVitty, 2015; Bond et al., 2020), and there still exists no generalized definition that covers all the facets it may cover. The concept of student engagement has attracted many researchers to debate or theorize it from the behavioral, psychological, sociocultural, and holistic perspectives (Kahu, 2013). The behavioral perspective emphasizes the influence of teaching practices on the behaviors of students, such as the time and the effort students devote to educational activities (Chickering and Gamson, 1987; Kahu, 2013). The psychological perspective regards engagement as an internal psychosocial process, enriching the definition of engagement with three dimensions: behavior (i.e., paralleling part of “behavioral perspective”), cognition (e.g., motivation, self-efficacy, and expectations) (Jimerson et al., 2003; Kahu, 2013), and affective dimensions (e.g., sense of belonging, enjoyment, and interest in tasks) (Furlong et al., 2003; Kahu, 2013). The sociocultural perspective focuses on the impact of social contexts and external factors (e.g., disciplinary power, academic culture, and the focus on performativity) on the experiences of students (Mann, 2001; Kahu, 2013). Some researchers have attempted to propose a holistic perspective, drawing these various perspectives together and taking the motivations and expectations of students into consideration (e.g., Yuan and Zhang, 2017). For example, “the three dimensions of student engagement” involve the emotional, cognitive, and behavioral engagement of students (Fredricks et al., 2004). “Emotional engagement” refers to the affective reactions of students to classroom activities (e.g., whether students enjoy the lessons) (Van Uden et al., 2014; Cents-Boonstra et al., 2020). “Cognitive engagement” indicates that students have their goals of learning and understanding the importance of education (Van Uden et al., 2014; Cents-Boonstra et al., 2020). “Behavioral engagement,” which might be active (e.g., asking questions) or passive (e.g., paying attention in class), means the extent to which behaviors of students are related to the learning process (Nguyen et al., 2018; Cents-Boonstra et al., 2020). Importantly, these three dimensions are interconnected.

Despite the different perspectives researchers may hold, they all agree that:

(1) the meaning of “student engagement” should be shaped by specific places and times (Ashwin and McVitty, 2015; Bond et al., 2020), which requires that teachers should analyze the external factors that might impact student engagement;
(2) student engagement is associated with student motivation (Yuan and Zhang, 2017; Bond et al., 2020; Cents-Boonstra et al., 2020) and persistence to learning (Archambault et al., 2009; Wang and Fredrick, 2014; Cents-Boonstra et al., 2020), which suggests that teachers should have a good understanding of internal factors of students (e.g., backgrounds, attitudes, confidence, and motivations of students) (Ferris et al., 2013; Ranalli, 2021);
(3) student engagement is subject to change over time, which indicates that student engagement can be either fostered or hindered in the learning environment in which they work (Cents-Boonstra et al., 2020);
(4) students who are engaged at school are likely to be engaged in their long-term vocation and will seek to attain occupational achievement (Abbott-Chapman et al., 2014; Cents-Boonstra et al., 2020), which foregrounds the importance of fostering student engagement.

These four agreements to some extent imply that teachers play a significant role in fostering student engagement, although it is admitted that students themselves are the persons who really decide to engage or disengage in learning (Shernoff et al., 2016; Quin et al., 2017; Cents-Boonstra et al., 2020; Harris and Leeming, 2021). Thus, both educational policymakers and researchers have been attracted to explore effective approaches to fostering student engagement.

Self-determination theory (SDT), for example, puts forward the premise that student engagement will be fostered when teachers support their three basic psychological needs (Van de Berghe et al., 2016; Cents-Boonstra et al., 2020): the need for autonomy, competence, and relatedness (Ryan and Deci, 2000; Cents-Boonstra et al., 2020). “The need for autonomy” is a sense of psychological freedom and volition to be oneself. To support this need, teachers are expected to invite students to provide suggestions for the teaching content (e.g., in the lesson plan, instructions, and learning activities) so that students can experience learning as a self-chosen act that reflects their own interests, preferences, and values (Stroet et al., 2013). “The need for competence” is the confidence students have that they can be successful academically (Cents-Boonstra et al., 2020), which can be supported by teachers providing students with appropriate and relevant learning experiences to enhance their self-efficacy (Bandura, 1995) (see details in the following section on self-efficacy). “Relatedness” refers to a close bond, which requires teachers to put enthusiasm into lessons, show an open, honest, and caring attitude toward students, and encourage students to support each other. Once students feel personally accepted and have a sense of belonging, positive teacher-student relationships can be developed and relatedness will be attained simultaneously (Korpershoek et al., 2019; Cents-Boonstra et al., 2020). To date, much of the research studies on student engagement have been conducted within the context of teaching and learning in offline classrooms.

Fostering student engagement in online classrooms, the new spaces, might be much more challenging. First, within online classroom students might come from diverse countries or regions, which means their sociocultural backgrounds might be quite different. Second, although students in online classrooms may have a greater sense of autonomy because of their self-chosen acts, they may also have more freedom to withdraw from learning; therefore, teachers need to invest more effort to foster the persistence and resilience of students (Asin, 1999). Third, it is challenging to attain relatedness online, because of limitations such as the teaching spaces, time, and internet connections. In addition, no matter what teaching platforms are used, there always exist some objective or subjective drawbacks or uncertainties that challenge teachers in the teaching process. The existing guidance of behaviors of teachers cannot solve these issues. Thus, it is necessary to deepen our understanding of student engagement online through the practices of teachers, and, understandably, the success of teachers in practices is largely related to their self-efficacy, to which we turn next.

### Self-Efficacy

Self-efficacy belief, also known as self-efficacy or efficacy belief (hereinafter “belief”), refers to “beliefs in one's capabilities to organize and execute the courses of action required to manage prospective situations” (Bandura, 1995, p. 2). Not only does self-efficacy influence the way people think and feel but also it motivates people to act and perform (Bandura, 1995). Therefore, people with a high level of self-efficacy have more motivation and persistence in performing tasks or adapting to changes in the professional field (Bandura, 2000, 2006; Morey and Ma, 2016). Self-efficacy is dynamic and may differ from one domain to the next (Bandura, 2006; Sela-Shayovitz and Finkelstein, 2020), and how it develops is influenced by four factors: mastery/performance experiences (personal authentic experience), vicarious experiences (authentic experiences of other people), social persuasion, and physiological and emotional state of an individual (Bandura, 1995; Bao et al., 2016; Chen and Zhang, 2019). Successful mastery or vicarious experiences and the positive climates of social or working contexts will work positively to increase self-efficacy, whereas failures may impact negatively (Bandura, 1995). However, whether an experience is a success or failure depends on the interpretation of an individual that is generated by a set of factors, such as personal, social, and situational ones (Bandura, 1995).

Based on this definition, numerous studies support that teaching is a process activated by the self-efficacy of teachers, the influence of which pervades before, during, and after the class. These studies have pointed out that three components make up a system of self-efficacy of teachers, including personal efficacy (beliefs and attitudes toward the teaching profession), efficacy within the organization (interpersonal relationships and support of the organization), and professional efficacy (the ability of individuals to carry out the tasks required in teaching) (Friedman and Kass, 2000; Wang et al., 2015; Sela-Shayovitz and Finkelstein, 2020). Each component interacts intricately with the experiences of a teacher (Bao et al., 2016), students, the organizational climate, and the quality of support provided by colleagues and the organization (Fives and Buehl, 2012; Hoffman and Seidel, 2015; Sela-Shayovitz and Finkelstein, 2020; Zhang, in press). Teachers, whose workplace is characterized by cooperation and support for teachers, tend to have a high sense of self-efficacy (Goddard and Goddard, 2001; Duran and Duran, 2005). These three components echo the three psychological needs (i.e., the needs for autonomy, relatedness, and competence) to foster student engagement (Ryan and Deci, 2000; Cents-Boonstra et al., 2020), which are also available for cultivating teacher engagement (Dutt et al., 2020): Personal efficacy supports the need of teachers for autonomy, efficacy within the organization for relatedness, and professional efficacy for competence. In turn, cultivated engagement of teachers will work on their self-efficacy.

Self-efficacy of teachers affects their long-term commitment to, engagement with, and persistence in the teaching profession, instructional decisions, and occupational well-being, which in turn impacts motivation, engagement, and learning of students (Pajares, 1992; Hofer and Pintrich, 1997; Klassen and Tze, 2014; Zee and Koomen, 2016; Lauermann and Berger, 2021). Teachers with high self-efficacy are more willing to set high teaching goals for themselves, experiment with new practices, and invest more effort in coping with difficulties (Dixon, 2011; Bao et al., 2016; Tan and Matsuda, 2020); they are also willing to support the development of intrinsic interests and self-directedness of students (Woolfolk and Hoy, 1990; Bandura, 1995), take responsibility for academic engagement, motivation, and achievements students, and be prepared for the failure of students (Goddard and Goddard, 2001; Guo et al., 2014; Sela-Shayovitz and Finkelstein, 2020).

However, recent research on self-efficacy in the Chinese context or Chinese students residing elsewhere has paid much attention to student learning (e.g., Lam and Chan, 2016), experiences of teacher burnout (e.g., Cao et al., 2018) and job satisfaction (e.g., Liu et al., 2018), emotions and experiences teachers of special education (e.g., Chen et al., 2020; Lu et al., 2020), and teachers' response to educational reforms and teacher identity (e.g., Zhang and Zhang, 2015; Huang et al., 2020). Very few research studies have focused on CFL teachers in the context of online teaching. This study thus sought to fill this gap with a case centering on the self-efficacy of a CFL teacher, exploring how she fostered online the engagement of Greek CFL students.

Informed by the three components of self-efficacy, the following three research questions (RQ) guided this study. These three questions also frame the themes (see *Findings*) emerging from the deductive data analysis.

RQ1: What are the teacher's beliefs about the opportunity of teaching CFL online? (Focusing on personal efficacy)
RQ2: What are the teacher's beliefs about the management of this online project? (Focusing on efficacy within the organization)
RQ3: What are the teacher's beliefs about her personal capability to foster students' engagement in this project? (Professional efficacy)

## Methods

Informed by the study of Dewey (1938) that “interaction” and “continuity” are the two principles of experience (Clandinin and Connelly, 2000), a narrative inquiry was adopted as a research methodology. In the field of language teaching, narrative inquiry, as a type of qualitative method, has proven to be an apt approach to investigate how language teachers interact with specific social, historical, and cultural contexts (Barkhuizen et al., 2014) and how the past experiences of teachers influence their teaching performances (Kane et al., 2002; Norman and Spencer, 2005; Casanave, 2012; Bao et al., 2016). These two principles are also consistent with “engagement in holistic perspective” that engagement is a dynamic continuum with different locations and times, which is best understood through in-depth qualitative study (Kahu, 2013).

### Research Context

This study is based on a project entitled *the Sino-Greece Online Chinese Classrooms*, which provides Chinese learners in Greece with a high-quality learning platform and rich learning resources, and aimed to promote cultural and educational exchanges between China and Greece. All the teachers involved in this project were provided with teaching materials such as prepared teaching plans and PowerPoint slides each week, which ensured that all the teachers followed the same curriculum and the same pace.

All the CFL teachers and teaching assistants (TAs) were selected from key universities in Mainland China. Teachers were the instructors of the course, while TAs assisted them to manage and observe classes and answered questions of students. Each TA assisted two teachers. Students enrolled in this project covered a diverse range of Greeks who were interested in learning Chinese. The students, for example, included both undergraduates and staff in a Greek University. The ages of the students varied from 20s to 50s. Because of pandemic lockdown, students took this part-time course in personal places rather than on the same campus. Hence, the effort of the teacher in motivating students to keep their enthusiasm for, and engagement in, learning was the prerequisite for the academic success of students.

The first semester of this project lasted 16 weeks: 13 weeks (1.5^*^2 h/ week) for teaching and 3 weeks for revision. After 16 weeks, students were expected to pass Level 1 of Hanyu Shuiping Kaoshi^2^ (HSK, a Chinese proficiency test with six levels; Level 1 is the lowest). That is to say, students should master 150 words/Chinese characters and their usages in daily conversation as required by the official HSK syllabus. Given that there existed no map directing how the online teaching and learning might occur, the completion of these tasks was challenging for both teachers and students. To this end, this study reports on a case study of a CFL teacher during the 16 weeks, focusing particularly on her self-efficacy beliefs about this project, to uncover how willing she was to undertake these tasks and how much effort she invested in teaching language skills and fostering student engagement.

### Participant: The CFL Teacher

The participant was CB, one of the frontline teachers of this project. She has a Ph.D. in Education (Applied Linguistics), focusing on language teaching and teacher development. Before this project, she had taught CFL in New Zealand (NZ) for 6 years. However, neither had she ever taught CFL online nor had she ever taught Greek students. She was thus faced with three main challenges: (1) teaching within an unfamiliar online teaching platform; (2) learning about Greek culture, an unfamiliar culture for her; and (3) catering for a diverse group of students in regard to age and prior learning experiences.

As both participant and researcher in this study, CB came back and forth between the two identities. As a participant, she was the central character who shared her lived and imagined experiences. As a researcher, she was sure to treat herself as the participant ethically; she was able to analyze and interpret the data fully, especially after negotiating with other researchers, and then made the illustrative data presented in findings closest to the lived reality of the participant.

### Data Collection

**Figure 1:**
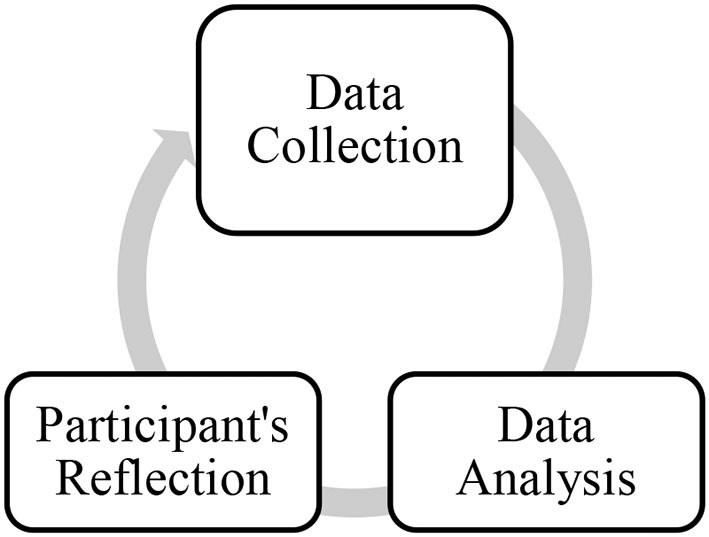
The process of data collection and data analysis.

Figure 1 indicates the research process, in which data collection is iteratively interwoven with data analysis (Dörnyei, 2007).

**Figure 2:**
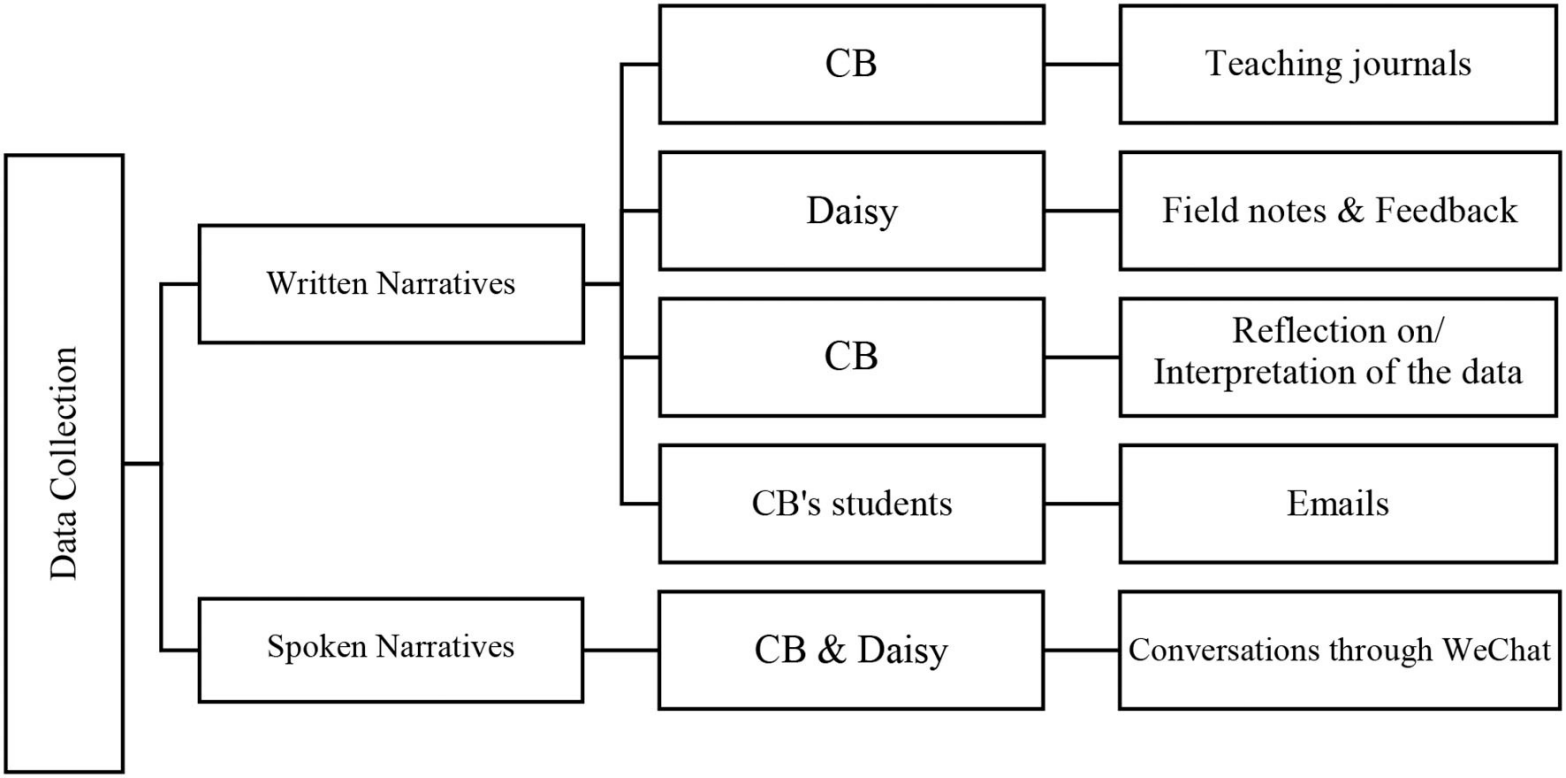
Data sources.

Following the process outlined by Polkinghorne (1995), this study used stories as research data (“analysis of narratives”) and storytelling as a tool for data analysis and presentation of findings (“narrative analysis”). The stories were mainly from two sources: written and spoken narratives (see Figure 2). The data from these sources were triangulated to enrich each other, mapping out a full picture of the self-efficacy of the teacher. As such, narrative inquiry in this study, to some extent, was CB's self-inquiry, the (re)constructive process (self-study and narrating) and product (enhanced knowledge and written/spoken narratives) of which enabled her to (re)interpret her experiences as a teacher and to build knowledge situated in her teaching context and with her students (Golombek and Johnson, 2017).

#### Written Narratives

Written narrative data consist of four sections: (1) “Teaching journals” were produced by CB during the 16 weeks, focusing on teaching strategies, emotions (e.g., fears, concerns, and desires), and the stories shared by students (Barkhuizen et al., 2014). These teaching journals might be written in her notebook or shared in the WeChat Moments (an online social networking service, similar to Facebook), in which some photographs were inserted. (2) “Field notes and feedback” were noted down by Daisy (pseudonym), a postgraduate student and TA of CB in this project. Her responsibility was to take notes of class observations (e.g., the behavior of teacher or students) and write down the feedback of students (e.g., thoughts, feelings, and comments of students online) for CB to read after class. (3) “Reflection on or interpretation of the data” was the stories shared by CB when she interpreted the narratives from the first two sections; these stories correlated with her previous experiences of teaching, learning, and living (Sakui, 2002), eliciting the causation of her perceived self-efficacy. (4) “Emails” were from the Greek students of CB, which in some sense worked as one of the builders of confidence of CB.

On the whole, CB wrote one time or two times a week while Daisy wrote one time every 2 weeks on average. The length of each entry was from several sentences to one or two handwritten pages. The emails were written by students occasionally. According to the timeline, the data were numbered (see details in Table 1). For example, TJ1 was written before TJ 2. All the data were originally in Chinese and translated by the researchers.

**Table 1:** Information of collected data.

**Sections**	**Providers**	**Types of data**	**Quantity**	**Coded numbers**
Written narratives	CB	Reflective teaching journals (TJ)	15	TJ 1–15
		The stories students shared (SS)	4	SS 1–4
		Unforgettable moments (UM)	4	UM 1–4
		Photos	7	Photos 1–7
	Daisy	Field notes and feedback	8	Daisy's Narratives 1–8
	CB's students	Emails after the project	5	(Student's pseudonym) Emails 1–5
Spoken narratives	CB and Daisy (transcribed by CB)	Conversations	6	Conversations 1–6

#### Spoken Narratives

Spoken narratives were from the conversations between CB and Daisy through the function of “Hold to talk” or “Voice Call” in WeChat. Conversations occurred when they needed to clarify the content in the field notes, talk about the performance of students, or discuss the topics related to CFL teaching and learning. These spoken narratives, which were transcribed by CB later, to some extent, enhanced the trustworthiness of the interpretation of the written narratives.

### Data Analysis

Thematic analysis was conducted deductively and inductively (Barkhuizen et al., 2014). With deductive thematic analysis, the three themes were determined in advance according to the three components of self-efficacy (Barkhuizen et al., 2014) (see Research Questions). With inductive thematic analysis, subthemes (see Findings) arose from data with constant contrast and comparison (Corbin and Strauss, 2008; Miles et al., 2014). Both the paths of thematic analysis were based on a repeated reading of the data and rounds of analysis so that the (sub)themes could be refined (Barkhuizen et al., 2014).

## Findings

### RQ1: Beliefs About This Opportunity of Teaching CFL Online (Focusing on Personal Efficacy)

The Chinese words below are what CB wrote for an official news report (published on February 21, 2021), indicating her robust self-efficacy about this opportunity.

Line 1 作为从教十余年的语言教师
*(Having been a language teacher for over 10 years)*
Line 2 确认自己是个幸运的人
*(I am sure that I am a lucky person)*
Line 3 行走在语言搭建的桥梁之上
*(With languages as bridges)*
Line 4 游走于不同的文化之间
*(I have a chance to know different cultures)*
Line 5 在与不同国家学生的交流中
*(and to communicate with students from different countries.)*
Line 6 感受自己的丰富与成长
*(I am really enjoying my life growing and becoming enriched in the process.)*
Line 7 曾经立志：“再奋斗二十年，让学生点亮世界地图。”
*(I once set a goal: “I am determined to work (teach CFL) another 20 years; then I will have taught students from all the countries in the world.”)*
Line 8 为了这个梦想，自己努力着、奋斗着、奔跑着。
  *For this goal, I have never stopped pursuing or endeavoring*.
Line 9 今年，点亮的国家是：希腊。
  *The students I will teach this year are from Greece*.
  (Excerpt from official news report, February 21, 2021)

From this news, it can be found that (1) she was proud of her identity as a language teacher (Lines 1–2); (2) she enjoyed intercultural communication, because teaching students from diverse cultural backgrounds also nourished herself in return (Lines 3–6); (3) she treated her career as a life-long mission, to which she was wholeheartedly devoted (Line 7–8); and (4) she was excited about having this precious opportunity to teach Greek students (Line 9). All the words and words between lines imply her firmly established beliefs that she loved her profession deeply, with no regrets.

In addition, “the moment of regaining the lost love” was closer to her emotion then. In her words:

“*Although I am enjoying the time with my students in China's University, I always miss the times when I taught CFL in New Zealand. In my present class, I sometimes shared some stories of my teaching in New Zealand…my present students were really interested in them (stories)…Whenever I heard foreigners speaking Chinese, I felt moved and excited… I am really proud of being a CFL teacher.”* (Excerpt from Reflection 1 of CB)

Notably, she missed the days of teaching CFL in NZ and her identity as a CFL teacher, so this opportunity supported her need for autonomy, reflecting her interests, preferences, and values (Stroet et al., 2013). She thus cherished this opportunity and undertook this teaching task without any hesitation.

### RQ2: Beliefs About the Management of This Online Project (Focusing on Efficacy Within the Organization)

The level of self-efficacy of CB within the organization was enhanced as the culture of collaboration in this project was being built (Sachs, 2005; Gong et al., 2018). Leaders of the online project created a supportive professional community by making continuous efforts to improve hardware facilities, providing teaching materials, and solving the problems encountered by either teachers or students instantly. Well-organized online meetings occurred regularly, encouraging teachers to communicate or share their experiences. Through these online meetings, teachers learnt from and helped each other. After the first meeting, CB stated:

*I have found a sense of belonging now… I love the community so much. We (teachers) encouraged each other and learned from each other…the meeting was so interesting…this kind of feeling is so cool!* (Excerpt from TJ 4)

However, when asked about her attitudes toward the organization before that meeting, she just replied that “I had no idea of it (this organization) …perhaps, neither liked nor disliked it” (Conversation 2). The change in the attitudes of CB reveals that it was after the meeting that CB started to become satisfied with the management of this project, because the atmosphere of the meeting brought to her a sense of belonging, and as such her need for relatedness was satisfied (Cents-Boonstra et al., 2020), which was essential to enhancing her relevant self-efficacy then.

### RQ3: Beliefs About Her Personal Capability to Foster Students' Engagement in This Project (Focusing on Professional Efficacy)

When CB was a preservice teacher, she was taught that a good lesson must encompass three indispensable constructs: knowledge to be taught, designs to be made, and students to be known. The three constructs were still what she firmly believed and stuck to. “Knowledge to be taught” refers to the knowledge chosen for a specific lesson, which is transferred from teacher to students implicitly or explicitly through “designs to be made” (i.e., the contents written in PowerPoint slides before the class, pedagogical teaching methods, and skills adopted during the class). “Students to be known” suggests that teachers should get familiar with the names, existing knowledge levels, and cultural backgrounds of the students before the class and further teacher-student relationship during and after the class, which is significant to support relatedness of both teachers and students. However, in a CFL class, the way to complete these three constructs cannot avoid being mediated by various challenges, because the knowledge of a second language is not only the “product of formal learning contexts, but it emerges out of the interaction of different social networks (family, cultural production, and school) with the individual cognitive and affective factors” (Menezes, 2008, p. 213); therefore, the latent challenges are worthy of the attention of researchers. As such, a series of challenges CB encountered in an effort to complete each construct emerged from the collected data. With inductive analysis, these challenges were summarized into nine themes within five categories in three periods: “teacher's preparation,” “selection of pedagogical methods and skills,” “cultural awareness,” “teacher-student relationship,” and “students' choices” (see details in Table 2). The intertwining of these challenges worked on the effects of CFL teaching of CB and the learning of students; whether the set teaching goals could be attained was largely dependent on her resilience to dwelling on, coping with, or balancing these challenges.

**Table 2:** Challenges in teaching.

**Time**	**Categories**	**Themes Summarized from the Data**	**Data resources**
Before the class	Teacher preparation	1. Teachers' capability of planning and reorganizing teaching materials	TJ 3
		2. Teachers lack knowledge about Greek culture and Greek language	TJ1; SS2
During the class	Selection of pedagogical methods and skills	3. Classroom languages: Chinese and English, because the space for TPR (Total Physical Response) is limited	TJ 5
		4. There are not many choices of in-class activities. It is impossible to do activities in out-of-class contexts	TJ 12; TJ 13
	Cultural awareness	5. Teachers should have cultural awareness	TJ 5
	Teacher-student relationship	6. Building teacher-student relationship in online class costs more time than in offline class	TJ 1; TJ 2; TJ 15
		7. Teaching process: Students consist of adult undergraduates and University teachers. They have their own understanding of the language; therefore, they liked to ask questions	TJ 13; TJ 14; TJ 15
After the class	Students' choices	8. To withdraw or to continue?	TJ6; SS 3
		9. To make effort to learn or to learn just for fun?	TJ 10

#### Preparation of Teacher (Before the Class)

Before the class, CB had a high degree of self-efficacy in relation to “knowledge to be taught” and “designs to be made,” which was reflected in her habit of reorganizing PowerPoint slides. To some extent, reorganizing slides also reflected her need for autonomy. As observed by Daisy, “the order of PowerPoint slides of Ms. CB is often different from the original one” (Narrative 3 of Daisy), to which CB explained,

*It is indeed a good way (using these teaching materials) to guarantee that all the teachers teach the same contents at the same pace. However, every teacher has his/her own thoughts, which will be reflected in the way he/she teaches. Actually, to reorganize the PowerPoint slides is to design my teaching plans*. (Excerpt from Reflection 3 of CB)

In contrast, the optimistic sense of her self-efficacy about “students to be known” was pessimistic. In the beginning, CB firmly believed that it was a must to memorize the names of the students. For one thing, calling the name of a person correctly can shorten the distance between two strangers, which is helpful to attain relatedness. For another, memorizing the names of other people is a way to show respect for others. Therefore, the teachers who can memorize the names of the students will be in the favor of students. This belief was initiated by her personal experiences as a high-school student. As she reflected,

*It is surprising that Ms. Tian (the name of her high-school teacher) could call my name when we met each other for the first time*. (Excerpt from Reflection 2 of CB)

When CB became a teacher, she thus took memorizing the names of the students in advance as her compulsory course, which was further reaffirmed by her own students. As one student told her,

*Ms. CB is the only teacher who can remember all the students' names within such short time*. (Excerpt from Reflection 2 of CB)

However, such confidence was completely diminished when she intended to memorize the official names of Greek students. As she said,

*I have a habit: I usually get familiar with students' names before I meet them. But now I don't think I can call their Greek names, which exceeds my coping capabilities. The knowledge of Greek language I know is only limited to some letters used in formulas of math or physics, such as* Ω,α,β,π,λ…*when these letters are put together, I have no idea of how to read at all, let alone the meanings of them. This kind of feelings is so bad*. (Excerpt from TJ 1)

It was thus noticed that in the first online lesson, she required all her students to register their English names on the screen and she never mentioned Greek names anymore, to which she explained:

*I tried to learn some Greek language online, but it was so difficult for me. I could not master Greek pronunciation within such short time, so I gave up*. (Excerpt from Reflection 3 of CB)

#### Selection of Pedagogical Methods and Skills (During the Class)

When selecting pedagogical methods and skills, CB realized that two main conflicts existed in this online project that affected her self-efficacy. While this was the case, she was determined to overcome the difficulties caused by the conflicts.

The first conflict was between teacher-centered and other approaches. Different network speeds caused asynchronism in the online teaching space, which determined that a teacher-centered approach was more appropriate than other approaches, such as the total physical response method (TPR) (Obitube et al., 2020) and the task-based language teaching method (TBLT) (Ellis et al., 2019). In addition, students were CFL beginners, whose Chinese language proficiency was not good enough to carry out complicated activities. From the perspective of psychological needs, this conflict was a challenge for the need of CB for autonomy. CB presented her reasons,

*I can only use English as a tool to explain the new knowledge, because it is impossible for students to understand explanation in Chinese. In addition, students only can see my face, shoulders, and hands on screen, so TPR is not available here*. (Excerpt from TJ 3)

In this sense, CB sometimes acted as a drill instructor helping students improve pronunciation and sometimes as an organizer guiding students to make up dialogues. All of the activities in class were student-student/teacher interactions.

The second conflict was between the aim of teaching and that of learning of students. The aim of teaching was to help students get familiar with Chinese and pass the HSK Level 1 (to get an official HSK certificate), so the teaching, to some extent, was examination-oriented. In contrast, all the students investigated in the class of CB expressed that they were only driven by curiosity and interest (e.g., one student can speak six languages: Greek, English, French, Italian, Spanish, and Portuguese). Therefore, what students were concerned with was only whether they had the chance to continue to learn rather than whether they could get an official certificate. In this way, the need of students for autonomy was challenged by teaching goals, the external factor. For example,

…*I would like to assure…if we don't take the exam, we can still have a chance to attend the lessons in the future*. (Excerpt from Email 2 of Cathy)

…*Our knowledge level is low so a certificate is really not useful…BUT I would really like to continue and this is my only concern*. (Excerpt from Email 2 of Anna)

Seemingly, the intrinsic interest and motivation of students fostered their learning performance. Their preference was not to accelerate learning to master more knowledge required in HSK Level 1, and they were more willing to talk about their favorite topics (e.g., how to express “I love you” and “handsome boys”) and to ask about the details they were curious about. “The students were so active and lovely. They learnt so hard for their interests” (observed by Daisy).

Although subjected to the two conflicts, CB confidently believed that she could exercise control over these challenges, which paid off in performance accomplishments (Bandura, 1995). Actually, it was her past experiences that supported her need for competence. She indeed made an endeavor to imbue her class with an active and relaxing atmosphere to arouse the desire of students for learning and balancing the required knowledge with the interests of students (Zhang and Zhang, 2020). The teaching outcomes proved that she not only achieved her teaching aims (e.g., all her students who attended HSK Level 1 passed it successfully) but also protected the interests of students in the Chinese language (e.g., students were eager to continue to learn). This successful experience in turn further strengthened her perceived self-efficacy in teaching approaches.

#### Cultural Awareness (During the Class)

Some researchers call for the integration of intercultural elements and cross-cultural awareness into CFL teacher educational programs (Moloney, 2013; Lai et al., 2015; Moloney and Xu, 2015; Gong et al., 2018). Indeed, cultural awareness is an essential skill for both language teachers and language learners, especially when they are in a community with diverse cultures. Any potential offense should be avoided. For language teachers, it is necessary to explain the cultural differences in advance when they communicate with students. For students, it is an effective way to master the target language through understanding cultural differences. In addition, the cultural awareness of both teachers and students helped to create an atmosphere filled with respect, so that the relatedness of every member of this class would be supported. Without any hesitation, the self-efficacy of CB was completely strong and she was well-prepared for intercultural teaching and for cultivating the intercultural competence of her students. As she wrote,

*When I taught numbers from 1 to 10, I asked students how to use hands to indicate these numbers. I found there were two biggest differences: (1) Greek students needed two hands if the number was bigger than 5; (2) When Greek students showed number 5, their palms of hands were facing themselves. Then I asked why they did in that way, they told me, the palms facing other people meant offending others*. (Excerpt from TJ 5)

She thus not only got the meaning of the special gesture then but also explained to the students at the same time,

*If you see Chinese people show hand with palm to you, you should also know that it is not intended offence…it is just because of the differences between us*. (Excerpt from TJ 5)

Henceforth, whenever she indicated five with hand, she would follow the Greek way. Apparently, CB had already raised her own cultural awareness. However, her cultural awareness was initiated incidentally when she taught in NZ. As she recalled the turning point,

*One of my Kiwi (the way local New Zealanders call themselves) students told me, “as a teacher, you should tell me how I am thinking; then I know the differences.” Since then, I have kept reminding myself of it*. (Excerpt from Reflection 2 of CB)

As some researchers pointed out, native Chinese language teachers educated in China are ill-prepared for intercultural teaching (Gong et al., 2018), which was also true for CB before that turning point occurred during her NZ teaching experiences.

#### Challenges of Teacher-Student Relationships (During the Class)

According to CB, the relationship between teachers and students online was quite different from that of offline; in other words, the relatedness online was more difficult to be supported. The online relationship was similar to net-friends, who would never meet in real life; the offline relationship was close to friends, who could make face-to-face interactions such as sharing experiences, singing Chinese songs, watching Chinese movies, and telling Chinese stories. Noticing these differences, CB invested effort to bridge the teacher-student distance psychologically *via* activities (e.g., dialogues) in class and emails after class, firmly believing:

*Although it was not easy (to build teacher-student relationship), I believed that we were all human beings who could feel empathy. Students could sense my care about them, especially when the sudden earthquake happened; in return, students said “xièxie (“Thank you” in Chinese)” to me, because what I cared about most was their safety then*. (Excerpt from Conversation 1)

The actions of CB resonated with her students. Even when the project was over, she still received some emails of students expressing “thank you, Ms. CB, for everything” (e.g., email of Elen).

In return, this sound teacher-student relationship also gave CB a sense of achievement. When CB was asked about which moment she felt happy as a teacher, she thought carefully and answered excitedly,

*When the students liked to share their life stories with me; when I could see students' smile on the screen; when students expressed their appreciation to me through emails and hoped to continue to study with me; when I was praised by administers…Then I knew I had been accepted by all. When I knew my students had passed HSK Level-1, I felt a sense of achievement. Then, I knew I had succeeded, because I had not only attained the teaching goals, but also satisfied students' learning need*. (Excerpt from Conversation 6)

As can be noticed in this narrative, although “administers' praise” encouraged CB, “students” were mentioned most. Apparently, the confidence and happiness of CB were tightly connected with the happiness, performance, and academic achievements of her students, which were also supported by the photographs she provided. One of the photographs, for example, was taken when students wrote Chinese characters successfully for the first time; in that picture, she and her students all smiled happily. She also used that picture in an official news report, hoping to share her happiness of that moment with the wider community.

#### Choices of Students (After the Class)

Teaching is like playing chess; both teachers and students are players, so both sides are important. Student engagement is influenced by both internal and external factors (Ferris et al., 2013; Ranalli, 2021). To warrant the quality of a language lesson, a language teacher must take into consideration the social networks of students (family, cultural production, and school) and cognitive and affective factors (Menezes, 2008) that might affect the choices of students, such as “to withdraw or to continue?” and “to make effort to learn or to learn just for fun?” presented in Table 2. The choices of students determine their dedication to learning, which in turn might influence teaching. Therefore, teachers need to sustain tenacious self-efficacy to exercise control over situations that are unmanageable (Bandura, 1995).

The first choice “to withdraw or to continue” originated from the characteristics of this project: a part-time course free of charge, which students selected voluntarily and could withdraw from any time. Taking a class of CB as an example, only 10 of the 18 registered students completed the course. Although CB had already understood that the students were adults and the decision they made must be reasonable, she, however, still felt depressed when students withdrew, especially when the withdrawal was caused by sudden and unexpected news. For example:

…*One student told me that he had lost her father that week, so he could not continue to follow the course anymore*. (Excerpt from SS1)

In addition to such personal stories, on October 30, 2020, when the second class of this course was being taught, a strong earthquake measuring 6.6 on the Richter scale hit Samos island of Greece in the eastern Aegean Sea according to the Institute of Geodynamics, the national observatory of Athens. As a result, some students had no choice but to give up learning. CB recalled:

*When the earthquake happened, I saw the shaking pictures on the screen, and then some students lost connection…That moment, what I only concerned was students' safety. I really hoped all of them could be safe. But I firmly believe that without earthquake, more students could continue to learn*. (Excerpt from Conversation 5)

After students chose to continue, the second choice came “to make effort to learn or to learn just for fun”: what learning attitudes they should hold and how much effort they would invest. After all, a traditional offline class and the current online class are two different learning environments, which in turn lead to different learning effects. A traditional offline classroom creates a relatively high-pressure learning atmosphere, in which students have to prepare well before each class so that they can master the knowledge well; teachers can guide and supervise students face-to-face. In contrast, an online classroom is a low-pressure learning community, which requires self-discipline and engagement of students if they hope to maintain progress (e.g., doing online homework instantly); teachers can only guide students to review orally; therefore, without the effort of students after class, it would be impossible for teachers to warrant teaching quality. In addition, all of the students (seven undergraduates; three staff in the Greek University) studied or worked at home during the lockdown, so their full-time work (e.g., full-time study, business of their families, and professional activities) kept them too busy. In such a situation, formal and concentrated language study became even more difficult for them. As CB wrote:

*After Christmas vacation, students have forgotten what they learnt before. Today, I had to help them to review and recall the knowledge they have already learnt, which led to the pace of teaching becoming much slower than I expected*. (Excerpt from TJ 14)

Despite these adversities, CB helped students review the learnt knowledge repeatedly and encouraged every student not to give up, which gave students more confidence and kept their engagement (Ferris et al., 2013; Ranalli, 2021). Eventually, both CB and her students attained their goals, even beyond their original expectations. The development of intrinsic interests of students also reaffirmed the strong self-efficacy of CB, with which she aroused the motivation of students, kept their engagement, and created mastery experiences for her students (Bandura, 1995). As one student expressed in the email,

*Dear Ms. CB*,

…*When I started I did not think I would continue due to my workload. I just started because I love languages. Thanks to you I kept up and I'm looking forward to learning more Chinese…* (Excerpt from Email 2 of Christy)

## Discussion

The findings show that CB, the CFL teacher, positively dealt with most of the challenges she encountered. Overall, she successfully sustained motivation for and engagement in learning of students and, eventually, helped them attain academic success. However, her self-efficacy in different (sub)themes was not only at dissimilar levels but also dynamic (Sela-Shayovitz and Finkelstein, 2020). To further analyze the factors relevant to the findings, CB was asked to fill in Table 3.

**Table 3:** Summary of findings.

**Self-efficacy**			**Levels of self-efficacy (Weakest) 1–5 (Strongest)**		**Influential factors**	
			**At the beginning**	**At the end**	**External factors (vicarious experiences, social persuasion, and working contexts and climates)**	**Internal factors (personal/mastery experiences, individual's physiological and emotional states)**
Personal efficacy			5	5	Support from colleagues and students (+)	Personal professional goal (+); Personal teaching experiences (+); Personal interests and enthusiasm (+); Sense of achievement (+)
Efficacy within the organization			3	5	Support from the organization and colleagues (+)	Sense of belonging (+)
Professional efficacy	Teacher's preparation	Knowledge to be taught; Teaching plans	5	5	Support from the organization and TA (+)	Personal teaching experiences (+); Knowledge of pedagogies (+)
		Students' names	5	2	Experience of CB's high-school teacher (+); Praise from CB's previous students (+); Greek language; (-)	Having no ability to master Greek language within such short time (–)
	Selection of teaching approaches		3	4	Limitations of the teaching space (–); Conflict between teacher-centered approach and other approaches (–); Conflict between the aim of teaching and that of students' learning (–)	Personal teaching experiences (+); Knowledge of pedagogies (+)
	Cultural awareness		5	5	Stories shared by CB's previous Kiwi student (+)	Personal teaching and learning experiences (+)
	Teacher-student relationship		2	4	Limitations of the teaching time and space (–); Appreciation from students (+)	Personal belief in the effects of communication and care (+); Sense of achievement (+)
	Students' choices	To withdraw or to continue?	2	2	Earthquake (–); Students' personal stories (–)	It was a pity (–)Sense of helplessness (–)
		To make effort to learn or to learn just for fun?	2	4	Students' personal stories (+/–)	Personal teaching experiences (+); Knowledge of pedagogies (+)

As reviewed, self-efficacy is a complicated system developed from four main factors: personal/mastery experiences, vicarious experiences, social persuasion, and the physiological and emotional states of an individual (Bandura, 1995). The collective effect of these factors makes it difficult to identify which one is more significant and effective in reality. Therefore, this study categorized the four factors into “external factors” and “internal factors” (see details in Table 3) and then analyzed how these factors jointly regulated the self-efficacy of CB.

In Table 3, the column of “Levels of self-efficacy” took Likert scale on a range from 1 to 5 (1 indicating the weakest beliefs, and 5 indicating the strongest beliefs) as a reference; the time of this column was divided into two sections: “at the beginning (of the project)” and “at the end (of the project),” so as to show the changes in beliefs of CB clearly. As the numbers in the two sections were presented, the changes in these beliefs were categorized into “enhanced,” “unchanged,” and “weakened.” The causal factors attributing to the changes were listed in the column of “influential factors,” including the external and internal factors that were summarized from the stories in “findings”; after that each factor was “+” or “-,” which indicates whether that factor played a positive or negative role, respectively.

### Enhanced Self-Efficacy Beliefs and Influential Factors

As Table 3 shows, the levels of four beliefs were enhanced, including beliefs in “efficacy within the organization,” “selection of teaching approaches,” “teacher-student relationship,” and “students' choices (to make effort to learn or to learn just for fun?).”

The influential factors in this group can be summarized as “negative external factors (in the beginning) and positive internal factors.” In particular, at the beginning of the project, the external factors contained information with many complexities, ambiguities, drawbacks, and uncertainties (e.g., new students and new teaching platform); therefore, CB should negotiate with students or adapt to the new teaching platform, which required effective cognitive processing (Bandura, 1995). Although the personal teaching experiences of CB (positive internal factors) reminded her that she had the capability to overcome the difficulties eventually, CB still needed more time and practice to enhance her confidence. CB thus drew on her knowledge to weigh and integrate predictive factors, to remember the effective factors she had tested, and to revise her actions (Bandura, 1995). Once she proved she could make it, her confidence was largely enhanced, then came a virtuous circle (Bandura, 2000, 2006; Morey and Ma, 2016). In this process, the self-efficacy of CB developed from weak to strong.

Taking “efficacy within the organization” as an example, CB did not reveal positive attitudes toward the project apparently until she felt cared for and supported by the leaders of this project. The positive feelings were reinforced after the first online meeting when she started to have a sense of belonging. The development of beliefs of CB was congruent with the previous study that the organizational climate and the quality of support provided by colleagues and organization will have positive effects on the self-efficacy of teachers (Goddard and Goddard, 2001; Duran and Duran, 2005; Sela-Shayovitz and Finkelstein, 2020). This also aligns with the sources of self-efficacy, which suggests that positive social persuasion and physiological and emotional states of an individual work positively on her self-efficacy (Bandura, 1995).

It should also be noted that her self-efficacy in “selection of teaching approaches” and “to make effort to learn or to learn just for fun?” that reached Level 4 after enhanced was still not the strongest. That was because the external factors were not positive then, in which situation what CB could do was to make the best of the existing conditions despite higher expectations she held for her personal teaching performance and academic engagement and achievement of students. In addition, among all the positive internal factors, the two mentioned most frequently were “personal teaching experience” and “sense of achievement,” both of which were closely interconnected with the support and encouragement from, and academic success achieved by, her students (see excerpts in *Findings*).

### Unchanged Self-Efficacy Beliefs and Influential Factors

Four self-efficacy beliefs stayed unchanged, three remained strong, and one weak. The three unchanged strong beliefs kept Level 5, including beliefs in “personal efficacy,” “efficacy in knowledge to be taught and making teaching plans,” and “efficacy in cultural awareness.” In this group, both external factors and internal factors were positive. Obviously, mastery experiences of CB of teaching and learning (positive internal factors) had already established her strong beliefs (Bandura, 1995); supported by positive external factors, she surely had enough confidence to solve the problems or overcome the difficulties (Dixon, 2011; Bao et al., 2016). For example, CB held robust beliefs about this teaching opportunity without any doubt, so she was devoted to this project and invested much effort to achieve her teaching goals and the academic success of students. Her strongest beliefs were also supported by her performances in “cultural awareness” and “preparing knowledge to be taught and making teaching plans.”

The only one unchanged weak belief stayed at Level 2, close to the weakest: “professional efficacy (the choice of the students: to withdraw or to continue?).” On the contrary to the condition of the three strongest beliefs, neither external factors nor internal factors were positive here when she faced the unmanageable situation, so “sense of helplessness” was her main feeling, which is echoed by the study of Bandura (1995) that “inability to exert influence over things that adversely affect one's life breeds apprehension, apathy, or despair” (p. 1).

### Weakened Self-Efficacy Beliefs and Influential Factors

Among the beliefs in all of the (sub) themes, only one was weakened: “professional efficacy (the names of the students).” Self-efficacy of CB in this aspect was at the highest level in the beginning when both external and internal factors were positive (e.g., the previous experiences of her teacher, students, and her own), but this belief was weakened when the negative external factor appeared (i.e., she realized the difficulty of Greek language). Although she invested effort to learn Greek pronunciation positively, the difficulty of the Greek language drowned her enthusiasm and undermined her confidence, so her need for competence could not be supported anymore. Distrusting her capability to master Greek pronunciation within such a short time slackened her effort (Bandura, 1995), so she disengaged in using Greek names of students finally and her belief concurrently became weaker (from Level 5 to Level 2).

## Conclusions

This narrative study has investigated the self-efficacy of a teacher in teaching CFL online and discussed relevant influential factors. By detecting the details of both external and internal factors, it provides a deep insight into the dynamic psychological states of a teacher as she faced a new teaching environment and made ongoing efforts to motivate students to engage in learning. As discussed above, self-efficacy beliefs of CB were mediated by the interplay of external factors and internal factors (see Table 4, the further summarized version of Table 3). When both of the factors were positive, her beliefs would keep strong and she would keep engaged; when both were negative, her beliefs would remain weak and she would disengage in the relevant act (e.g., learning Greek names). When external factors were filled with uncertainties or drawbacks, the internal factors would be more important in her performance and the development of self-efficacy beliefs.

**Table 4:** Summary of discussion.

**External factors**	**Internal factors**	**Change of self-efficacy beliefs**
+	+	Unchanged strong
–	–	Unchanged weak
–	+	Enhanced
+	–	Not mentioned

It should be noted that there is no actual standard criterion to judge whether an external factor is positive or negative; in effect, the judgment was made by CB subjectively. Positive external factors, in her opinion, gave her a sense of security or belonging (i.e., relatedness), which thus led her to develop a relatively strong self-efficacy and more engagement. Those negative ones were new factors that appeared in this project. In order to cope with the new factors that she perceived as drawbacks or uncertainties, she needed to strengthen or reestablish her self-efficacy (i.e., to support the need for competence) through more practices even if her established self-efficacy was already strong. In effect, these findings are also resonated with the “broaden and build” theory in positive psychology (Fredrickson, 2005), which emphasizes the following: Emotion is a precondition for career exploration (Fredrickson, 2005; Robertson, 2018); people can establish positive emotion by engaging in purposeful or prosocial activities, fulfilling their true nature and effective functioning (Ryan et al., 2008); and established positive emotion will lead to a virtuous circle of learning and development in career (Fredrickson, 2005; Robertson, 2018).

In addition, both CFL and online teaching are becoming increasingly popular in the world; therefore, how to keep academic motivation, engagement, and achievements of CFL students online calls for more attention from teachers and researchers. As supportive materials, the stories presented in this study provided a picture of the Sino-Greece online CFL project, including its organization, the status of enrolled students, the challenges teachers might encounter, the goals of teaching of teachers and learning of students, and the achievement it might attain. This picture might be a map for future online teaching in CFL or other similar contexts.

This study was conducted at an individual level. Admittedly, the findings from a single case study cannot be generalized, but these findings also have implications for the questions at hand (Flyvbjerg, 2006; Gao, 2010). At present, online teaching, as a teaching approach, is being shared by an increasing number of teachers around the world; hence, it is reasonable to assume that the experiences of CB and the trajectories of her self-efficacy share some similarities with those in the cases of other teachers, which can be tested through further research studies at a collective level in the future.

## Data Availability Statement

The original contributions generated for the study are included in the article/supplementary material, further inquiries can be directed to the corresponding author/s.

## Ethics Statement

Ethical review and approval was not required for the study on human participants in accordance with the local legislation and institutional requirements. The patients/participants provided their written informed consent to participate in this study.

## Author Contributions

CB conceived and designed the study, collected and analyzed the data, and drafted the first manuscript. LZ and HD revised the manuscript. All authors agreed to the final version before LZ got it ready for submission.

## Conflict of Interest

The authors declare that the research was conducted in the absence of any commercial or financial relationships that could be construed as a potential conflict of interest.

## Publisher's Note

All claims expressed in this article are solely those of the authors and do not necessarily represent those of their affiliated organizations, or those of the publisher, the editors and the reviewers. Any product that may be evaluated in this article, or claim that may be made by its manufacturer, is not guaranteed or endorsed by the publisher.

## Footnotes

^1^From “Ministry of Education: The Number of Foreigners Learning Chinese Exceeds 20 Million” (June 5, 2021) (http://www.chinese.cn/page/#/pcpage/article?id=699&page=1).

^2^HSK (Level I) assesses the abilities of test-takers in the application of everyday Chinese. It is the counterpart of the Level I of the *Chinese Language Proficiency Scales for Speakers of Other Languages* and the A1 Level of the *Common European Framework of Reference (CEF)*. Test-takers who are able to pass the HSK (Level I) can understand and use very simple Chinese phrases, meet basic needs for communication, and possess the ability to further their Chinese language studies (from http://www.chinesetest.cn/gosign.do?id=1&lid=0#).
